# Echoes of solitude: systematic review and meta-analysis revealing mortality risks in older adults due to loneliness, social isolation, and living alone

**DOI:** 10.1192/j.eurpsy.2025.1746

**Published:** 2025-08-26

**Authors:** A. Nakou, E. Dragioti, N. Zagorianakou, F. Tatsis, S. Mantzoukas, M. Gouva

**Affiliations:** 1 Department of Nursing, School of Health Sciences, University of Ioannina, Ioannina, Research Laboratory Psychology of Patients, Families & Health Professionals; 2 Department of Nursing, School of Health Sciences, University of Ioannina, Ioannina, Research Laboratory of Integrated Health, Care and Well-being, Ioannina, Greece

## Abstract

**Introduction:**

Loneliness, social isolation, and living alone are emerging as significant risk factors for mortality, especially in older adults.

**Objectives:**

Loneliness, social isolation, and living alone are recognized as significant risk factors for mortality in older adults. This study aimed to quantify their associations with all-cause and cause-specific mortality, extending the scope of previous research by considering a broader range of social factors.

**Methods:**

A systematic search was conducted in PubMed, APA PsycINFO, and CINAHL databases up to December 31,2023, adhering to PRISMA 2020 and MOOSE guidelines. Inclusion criteria comprised prospective cohort or longitudinal studies examining the relationship between loneliness, social isolation, living alone, and mortality. Quality assessment was performed using the Newcastle-Ottawa Scale. Meta-analyses utilized random-effects models with the Restricted Maximum Likelihood method, while subgroup and meta-regression analyses explored further relationships.

**Results:**

Out of 11,964 studies screened, 86 met the inclusion criteria. Loneliness was associated with a 14% increase in all-cause mortality risk, social isolation with a 35% increase, and living alone with a 21% increase. However, substantial heterogeneity was observed across studies, influenced by various factors including gender, age, geographical region, chronic diseases, and study quality. Meta-regression analysis identified predictors such as longer follow-up periods, female sex, validated social network indices, cognitive function adjustments, and study quality.

**Conclusions:**

Loneliness, social isolation, and living alone significantly increase mortality risk in older adults, emphasizing the urgency of public health interventions targeting these factors to enhance health outcomes among the aging population. However, due to study variations, further research is needed to understand their cumulative effect on mortality risks and inform tailored interventions.

**Disclosure of Interest:**

None Declared

